# Supplementary material for: Assisted Reproductive Technique and Abnormal Cord Insertion: A Systematic Review and Meta-Analysis
Source: Biomedicines. 2022 Jul 17;10(7):1722. doi: 10.3390/biomedicines10071722 (PMC9312931; doi:10.3390/biomedicines10071722)
Supplement: Supplementary file 1 [file biomedicines-10-01722-s001.zip › biomedicines-1773205-supplementary.pdf]

Supplemental File S1. The search strategy.

PubMed

#1 “In Vitro Fertilization” [TIAB] OR fertilization in vitro [MeSH] OR Assisted Reproductive Techniques [MeSH] OR Embryo Transfer [MeSH] OR Intracytoplasmic Sperm Injection [MeSH] OR Cryopreserved [TIAB] OR "Oocyte donation" [TIAB] OR "fresh cycle" [TIAB] OR "frozen cycle" [TIAB] OR cleavage [TIAB] OR blastocyst [TIAB]

#2 Umbilical cord [MeSH]

#3 velamentous [TIAB]

#4 abnormal cord insertion [TIAB] OR marginal cord insertion [TIAB]

#5 vasa previa [TIAB]

#6 membranous fetal vessel [TIAB] OR membranous cord [TIAB]

#7 #2 OR #3 OR #4 OR #5 OR #6

#8 #1 AND #7

#9 probability [TIAB] OR likelihood [TIAB] OR odds ratio [TIAB] OR risk ratio [TIAB] OR risk assessment [TIAB] OR logistic [TIAB] OR regression [TIAB] or multivaria* [TIAB] OR univaria* [TIAB]

#10 #7 AND #9

#11 #8 OR #10

Cochrane

#1 “In Vitro Fertilization”:ab,ti,kw

#2 MeSH descriptor: [fertilization in vitro] explode all trees

#3 MeSH descriptor: [Assisted Reproductive Techniques] explode all trees

#4 MeSH descriptor: [Embryo Transfer] explode all trees

#5 MeSH descriptor: [Intracytoplasmic Sperm Injection] explode all trees

#6 Cryopreserved:ab,ti,kw

#7 "Oocyte donation":ab,ti,kw

#8 "fresh cycle":ab,ti,kw

#9 "frozen cycle":ab,ti,kw

#10 cleavage:ab,ti,kw OR blastocyst:ab,ti,kw

#11 #1 OR #2 OR #3 OR #4 OR #5 OR #6 OR #7 OR #8 OR #9 OR #10

#12 velamentous :ab,ti,kw

#13 “abnormal cord insertion”:ab,ti,kw

#14 “marginal cord insertion”:ab,ti,kw

#15 “vasa previa”:ab,ti,kw

#16 “membranous fetal vessel”:ab,ti,kw OR “membranous cord”:ab,ti,kw

#17 #12 OR #13 OR #14 OR #15 OR #16

#18 #11 AND #17

#19 probability:ab,ti,kw OR likelihood:ab,ti,kw OR odds ratio:ab,ti,kw OR risk ratio:ab,ti,kw OR risk assessment:ab,ti,kw OR logistic:ab,ti,kw OR regression:ab,ti,kw or multivaria*:ab,ti,kw OR inivaria*:ab,ti,kw

#20 #17 AND #19

#21 #18 OR #20

Scopus

#1 TITLE-ABS-KEY ("In Vitro Fertilization" OR "fertilization in vitro" OR "Assisted Reproductive Techniques" OR "Embryo Transfer" OR "Intracytoplasmic Sperm Injection" OR "Cryopreserved " OR "oocyte donation" OR "fresh cycle" OR "frozen cycle" OR "cleavage” OR “blastocyst”)

#2 TITLE-ABS-KEY ("velamentous" OR “marginal cord insertion” OR “abnormal cord insertion” OR "vasa previa” OR membranous fetal vessel*” OR “membranous cord”)

#3 #1 AND #2

#4 TITLE-ABS-KEY (probability OR likelihood OR odds ratio OR risk ratio OR risk assessment OR logistic OR regression OR multivaria* OR univaria*)

#5 #2 AND #4

#6 #3 OR #5

Supplemental Table S1. The definition of heterogeneity.

I^2 value	Heterogeneity	Analysis
0%–<30%	Low	Fixed-effect
30%–60%	Moderate	Random-effect
50%–90%	Substantial	Random-effect
75%–100%	Considerable	Random-effect

According to the *Cochrane Handbook for Systematic Reviews of Interventions* (ver 6.0), heterogeneity was determined per the I^2 value [1].

Supplemental Table S2. Risk of bias assessment for the comparator study.

Authors	Confounding	Selection	Classification of intervention	Deviations from interventions	Missing data	Measurement of outcomes	Reported results	Overall bias
Furuya S [2]	Moderate	Serious	Serious	No information	Moderate	Moderate	Moderate	Serious
Swanson K [3]	Moderate	Serious	Serious	No information	No information	Moderate	Moderate	Serious
Volodarsky A [4]	Moderate	Serious	Serious	No information	No information	Moderate	Moderate	Serious
Sacha CR [5]	Moderate	Serious	Serious	No information	No information	Moderate	Moderate	Serious
Yang M [6]	Moderate	Moderate	Moderate	Moderate	No information	Moderate	Moderate	Moderate
Cochrane E [7]	Moderate	Serious	Serious	Moderate	No information	Moderate	Moderate	Moderate
O'Quinn C [8]	Moderate	Moderate	Moderate	Moderate	Low	Moderate	Moderate	Moderate
Yanaihara A [9]	Moderate	Moderate	Moderate	Moderate	No information	Moderate	Moderate	Moderate
Kalafat E [10]	Moderate	Serious	Moderate	Moderate	No information	Moderate	Moderate	Serious
Ebbing C [11]	Moderate	Moderate	Moderate	Moderate	Serious	Moderate	Moderate	Moderate
Suzuki S [12]	Moderate	Moderate	Moderate	Moderate	No information	Moderate	Moderate	Moderate
Ebbing C [13]	Moderate	Moderate	Moderate	Moderate	No information	Moderate	Moderate	Moderate
Räisänen S [14]	Moderate	Moderate	Moderate	Moderate	No information	Moderate	Moderate	Moderate
Delbaere I [15]	Moderate	Serious	Moderate	Moderate	Serious	Moderate	Moderate	Moderate
Baulies S [16]	Moderate	Moderate	Moderate	Moderate	No information	Moderate	Moderate	Moderate
Jauniaux E [17]	Moderate	Serious	Moderate	Moderate	Moderate	Moderate	Moderate	Serious

Risk of bias assessment was performed using the Risk Of Bias In Non-randomized Studies–of Interventions tool (ROBINS-I) [18-20].

● Low risk of bias (the study is comparable to a well-performed randomized trial with regard to this domain)

● Moderate risk of bias (the study is sound for a non-randomized study with regard to this domain but cannot be considered comparable to a well-performed randomized trial)

● Serious risk of bias (the study has some important problems in this domain)

● Critical risk of bias (the study is too problematic in this domain to provide any useful evidence on the effects of intervention.

● No information on how to base a judgment on the risk of bias for this domain.

Supplemental Table S3. Metadata of included studies.

Author	Year	Location	Total No.	Matched	Control No.	ART No.	Cont_VCI	ART_VCI	Cont_MCI	ART_MCI	Cont_VP	ART_VP
Furuya S [2]	2021	JPN	906	No	0	906 (100%)	0	55	--	--	--	--
Swanson K [3]	2021	USA	311	No	0	311 (100%)	0	14	--	20	--	--
Volodarsky A [4]	2021	CAN	677	No	0	677 (100%)	0	54	0	166	--	--
Sacha CR [5]	2020	USA	1140	No	0	1140 (100%)	0	86	0	138	--	--
Yang M [6]	2020	CHN	59976	No	58351	1625 (2.7%)	482	19	--	--	--	--
Cochrane E [7]	2020	USA	360	Yes	240	120 (33.3%)	7	14	35	34	--	--
O'Quinn C [8]	2020	CAN	32487	No	24992	1524 (4.7%)	83	5	1066	116	--	--
Yanaihara A [9]	2018	JPN	1610	No	1453	157[#] (9.8%)	145	32	--	--	--	--
Kalafat E [10]	2018	GBR	497*	No	338	159 (32.0%)	32	18	125	45	--	--
Ebbing C[†] [11]	2017	NOR	860465	No	843531	16934 (2.0%)	12422	589	47685	1475	--	--
Suzuki S [12]	2015	JPN	16965	No	16029	936 (5.5%)	132	36	--	--	--	--
Ebbing C[†] [13]	2013	NOR	623478	No	613270	10208 (1.6%)	9127	373	38442	961	--	--
Räisänen S [14]	2012	FIN	26849	No	26172	677 (2.5%)	611	22	--	--	--	--
Delbaere I [15]	2007	BEL	4159*	No	2119	880 (21.2%)	76	65	184	125	--	--
Baulies S [16]	2007	SPN	12063	No	11239	824 (6.8%)	--	--	--	--	5	4
Jauniaux E [17]	1990	GBR	100	Yes	50	50 (50.0%)	1	6	5	13	--	--

* all cases were twins. [#] All embryos were frozen and transferred on day 5 (blastocyst stage). [†] Used same database.

Abbreviations: No., number of included cases; Matched, patient background matched study; ART, assisted reproductive technology; VCI, velamentous cord insertion; Control_VCI, number of women with velamentous cord insertion in control group; Cont_VP, number of women with vasa previa in control group; ART_VCI, number of women with velamentous cord insertion in ART group; ART_VP, number of women with vasa previa in control group; CD, cesarean delivery; ID, instrumental deivery, SGA, small for gestational age; and PAS, placenta accreta spectrum.

Supplemental Table S4. The effect of abnormal cord insertion on the rate of cesarean delivery.

Author	Year	Cont No.	VCI No.	Cont_CD	VCI_CD	Cont_el CD	VCI_el CD	Cont_em CD	VCI_em CD	Cont_ID	VCI_ID
Furuya S [2]	2021	851	55	--	--	--	--	174	23	158	8
Yang M [6]	2020	59475	501	9456	116	4163	35	5293	81	--	--
O'Quinn C [8]	2020	30953	107	8367	40	--	--	--	--	4871	16
Suzuki S [12]	2015	16797	168	--	--	--	--	1439	29	--	--
Ebbing C [13]	2013	613978	9500	82949	1946	34119	600	48330	1346	50778	783
Räisänen S [14]	2012	26216	633	4064	140	1783	43	2281	97	2124	56
Author	Year	Cont No.	MCI No.	Cont_CD	MCI_CD	Cont_el CD	MCI_el CD	Cont_em CD	MCI_em CD	Cont_IDD	MCI_ID
O'Quinn C [8]	2020	30953	1427	8367	388	--	--	--	--	4871	224
Ebbing C [13]	2013	584075	39403	78037	6858	32121	2598	45916	4260	48316	3245

Abbreviations: Cont No., number of women in control group; Cont_CD, number of cesarean deliveries in control group; el CD, elective cesarean delivery; em CD, emergent cesarean delivery; ID, instrumental delivery; VCI No., Number of women with velamentous cord insertion; MCI No., number of women with marginal cord insertion; VCI_CD, Number of cesarean deliveries in velamentous cord insertion group; MCI_CD, number of cesarean deliveries in marginal cord insertion group.

Supplemental Table S5. The effect of MCI on the delivery outcome.

Author	Year	Cont No.	Cont events	MCI No.	MCI events	Crude OR (95%CI)	Adjusted OR (95%CI)
CD (all)							
O'Quinn C [8]	2020	30953	8367	1427	388	1.01 (0.89-1.14)	--
Ebbing C [13]	2013	584075	78037	39403	6858	1.37 (1.33-1.40)	--
Elective CD							
Ebbing C [13]	2013	584075	32121	39403	2598	1.21 (1.16-1.26)	--
Emergent CD							
Ebbing C [13]	2013	584075	45916	39403	4260	1.42 (1.37-1.47)	--
Instrumental delivery							
O'Quinn C [8]	2020	30953	4871	1427	224	1.00 (0.86-1.15)	--
Ebbing C [13]	2013	584075	48316	39403	3245	1.00 (0.96-1.03)	--

We calculated ORs using RevMan version 5.4.1 and some values were inferred by authors; thus, certain values in the table be slightly different from the original values. Abbreviations: Cont No., number of women in control group; Cont events, number of events in control group; VCI No., Number of women with marginal cord insertion; MIC events, Number of events in marginal cord insertion group; Crude OR, crude odds ratio; Adjusted OR, adjusted odds ratio; CI, confidence interval.

Supplemental Table S6. The relationship between VCI and abnormal placentation.

Author	Year	Control	VCI No.	Cont previa	VCI previa	Cont PASD	VCI PASD
Furuya S [2]	2021	851	55	--	--	16	4
Yang M[6]	2020	59475	501	357	8	--	--
Suzuki S [12]	2015	16797	168	164	5	97	3
Ebbing C [13]	2013	613978	9500	1713	102	--	--
Räisänen S [14]	2012	26216	633	184	11	--	--
Author	Year	Control	MCI No.	Cont previa	MCI previa	Cont PAS	MCI PAS
Ebbing C [13]	2013	584075	39403	1614	201	--	--

Abbreviations: Cont No., number of women in control group; VCI No., Number of women with velamentous cord insertion; MCI No., Number of women with marginal cord insertion; VCI, velamentous cord insertion; MCI, magrinal cord insertion; Cont, control; PASD, placenta accreta spectrum of disorder.

References

1. Cochrane Handbook for Systematic Reviews of Interventions. Version 6.1, 2020. Chapter 10: Analysing data and undertaking meta-analyses. https://training.cochrane.org/handbook/current/chapter-10. (accessed 18/12/2020).
2. Furuya, S.; Kubonoya, K.; Yamaguchi, T. Incidence and risk factors for velamentous umbilical cord insertion in singleton pregnancies after assisted reproductive technology. *The journal of obstetrics and gynaecology research* **2021**, *47*, 1772-1779, doi:10.1111/jog.14727.
3. Swanson, K.; Huang, D.; Kaing, A.; Blat, C.; Rosenstein, M.G.; Mok-Lin, E.; Gras, J.; Sperling, J.D. Is Preimplantation Genetic Testing Associated with Increased Risk of Abnormal Placentation After Frozen Embryo Transfer? *American journal of perinatology* **2021**, *38*, 105-110, doi:10.1055/s-0040-1714681.
4. Volodarsky-Perel, A.; Ton Nu, T.N.; Buckett, W.; Machado-Gedeon, A.; Cui, Y.; Shaul, J.; Dahan, M.H. Effect of embryo stage at transfer on placental histopathology features in singleton live births resulting from fresh embryo transfers. *Fertility and sterility* **2021**, *115*, 673-682, doi:10.1016/j.fertnstert.2020.06.017.
5. Sacha, C.R.; Harris, A.L.; James, K.; Basnet, K.; Freret, T.S.; Yeh, J.; Kaimal, A.; Souter, I.; Roberts, D.J. Placental pathology in live births conceived with in vitro fertilization after fresh and frozen embryo transfer. *American journal of obstetrics and gynecology* **2020**, *222*, 360.e361-360.e316, doi:10.1016/j.ajog.2019.09.047.
6. Yang, M.; Zheng, Y.; Li, M.; Li, W.; Li, X.; Zhang, X.; Wang, R.; Zhang, J.; Zhou, F.; Yang, Q.; et al. Clinical features of velamentous umbilical cord insertion and vasa previa: A retrospective analysis based on 501 cases. *Medicine* **2020**, *99*, e23166, doi:10.1097/md.0000000000023166.
7. Cochrane, E.; Pando, C.; Kirschen, G.W.; Soucier, D.; Fuchs, A.; Garry, D.J. Assisted reproductive technologies (ART) and placental abnormalities. *Journal of perinatal medicine* **2020**, *48*, 825-828, doi:10.1515/jpm-2020-0141.
8. O'Quinn, C.; Cooper, S.; Tang, S.; Wood, S. Antenatal Diagnosis of Marginal and Velamentous Placental Cord Insertion and Pregnancy Outcomes. *Obstetrics and gynecology* **2020**, *135*, 953-959, doi:10.1097/aog.0000000000003753.
9. Yanaihara, A.; Hatakeyama, S.; Ohgi, S.; Motomura, K.; Taniguchi, R.; Hirano, A.; Takenaka, S.; Yanaihara, T. Difference in the size of the placenta and umbilical cord between women with natural pregnancy and those with IVF pregnancy. *Journal of assisted reproduction and genetics* **2018**, *35*, 431-434, doi:10.1007/s10815-017-1084-2.
10. Kalafat, E.; Thilaganathan, B.; Papageorghiou, A.; Bhide, A.; Khalil, A. Significance of placental cord insertion site in twin pregnancy. *Ultrasound in obstetrics & gynecology : the official journal of the International Society of Ultrasound in Obstetrics and Gynecology* **2018**, *52*, 378-384, doi:10.1002/uog.18914.
11. Ebbing, C.; Johnsen, S.L.; Albrechtsen, S.; Sunde, I.D.; Vekseth, C.; Rasmussen, S. Velamentous or marginal cord insertion and the risk of spontaneous preterm birth, prelabor rupture of the membranes, and anomalous cord length, a population-based study. *Acta obstetricia et gynecologica Scandinavica* **2017**, *96*, 78-85, doi:10.1111/aogs.13035.
12. Suzuki, S.; Kato, M. Clinical Significance of Pregnancies Complicated by Velamentous Umbilical Cord Insertion Associated With Other Umbilical Cord/Placental Abnormalities. *Journal of clinical medicine research* **2015**, *7*, 853-856, doi:10.14740/jocmr2310w.
13. Ebbing, C.; Kiserud, T.; Johnsen, S.L.; Albrechtsen, S.; Rasmussen, S. Prevalence, risk factors and outcomes of velamentous and marginal cord insertions: a population-based study of 634,741 pregnancies. *PloS one* **2013**, *8*, e70380, doi:10.1371/journal.pone.0070380.
14. Räisänen, S.; Georgiadis, L.; Harju, M.; Keski-Nisula, L.; Heinonen, S. Risk factors and adverse pregnancy outcomes among births affected by velamentous umbilical cord insertion: a retrospective population-based register study. *European journal of obstetrics, gynecology, and reproductive biology* **2012**, *165*, 231-234, doi:10.1016/j.ejogrb.2012.08.021.
15. Delbaere, I.; Goetgeluk, S.; Derom, C.; De Bacquer, D.; De Sutter, P.; Temmerman, M. Umbilical cord anomalies are more frequent in twins after assisted reproduction. *Human reproduction (Oxford, England)* **2007**, *22*, 2763-2767, doi:10.1093/humrep/dem191.
16. Baulies, S.; Maiz, N.; Muñoz, A.; Torrents, M.; Echevarría, M.; Serra, B. Prenatal ultrasound diagnosis of vasa praevia and analysis of risk factors. *Prenatal diagnosis* **2007**, *27*, 595-599, doi:10.1002/pd.1753.
17. Jauniaux, E.; Englert, Y.; Vanesse, M.; Hiden, M.; Wilkin, P. Pathologic features of placentas from singleton pregnancies obtained by in vitro fertilization and embryo transfer. *Obstetrics and gynecology* **1990**, *76*, 61-64.
18. Sterne, J.A.; Hernan, M.A.; Reeves, B.C.; Savovic, J.; Berkman, N.D.; Viswanathan, M.; Henry, D.; Altman, D.G.; Ansari, M.T.; Boutron, I.; et al. ROBINS-I: a tool for assessing risk of bias in non-randomised studies of interventions. *BMJ* **2016**, *355*, i4919, doi:10.1136/bmj.i4919.
19. Danna, S.M.; Graham, E.; Burns, R.J.; Deschenes, S.S.; Schmitz, N. Association between Depressive Symptoms and Cognitive Function in Persons with Diabetes Mellitus: A Systematic Review. *PLoS One* **2016**, *11*, e0160809, doi:10.1371/journal.pone.0160809.
20. ROBINS-I detailed guidance (2016). https://www.riskofbias.info/welcome/home/current-version-of-robins-i/robins-i-detailed-guidance-2016. (accessed 09/20/2020).
